# A Strain Feedback Compensation Method during Cell Tensile Experiments

**DOI:** 10.1155/2017/1587670

**Published:** 2017-06-18

**Authors:** Rong Zhou, Yunshu Yang, Wenzhuo Zhang, Yuanwen Zou

**Affiliations:** College of Materials Science and Engineering, Sichuan University, Chengdu 610065, China

## Abstract

Cell tensile technique is an important and widely used tool in cell mechanical research. However, the strain control condition in traditional tensile experiments is not satisfied and would result in big errors. These strain errors will seriously impact the experimental accuracy and decrease the reliability and comparability of experimental results. In order to achieve the accurate strain control of the membrane during stretching, a strain feedback compensation method based on the digital image correlation is proposed in this paper. To evaluate the effect of the proposed compensation method, a series of stretching experiments in different strains ranging from 5% to 20% were performed. The results showed that our proposed method significantly decreased the errors of strain control. These results indicate that the strain feedback compensation method is very effective in controlling strain and can greatly improve the experimental accuracy.

## 1. Introduction

Biomechanical stimuli have been utilized to enhance the growth, strength, and functionality of engineered tissues [1, 2]. At the cellular level, mechanical stretching could vitally control over cell morphology, proliferation, lineage commitment, and differentiation [3, 4]. Cell tensile technique is a fundamental method to exert tension on cells. The cells adhered to the membrane would follow its deformation and thereby experience tensile strain. Obviously, the strain control of the membrane is of great importance. In traditional tensile experiments, operators calculate the moving distance of the clamps according to the initial clamping length of the membrane and the desired strain and then use it to control the motors. However, the actual strain condition of this method is not satisfied. Zhang et al. [5] proposed that the clamp-to-clamp strain was greater than the actual measured strain because the indicated displacement included the sliding of the specimen within clamps. Colombo et al. [6] analyzed the strain field of Flexcell system for different waveforms and frequencies and demonstrated that the measured strains of membranes showed notable differences between both the inputs and the outputs of the Flexcell software. Riehl et al. [7] summarized many types of cell tensile devices and concluded that the strain conducted to the membrane is affected by numerous factors, including membrane properties, clamping method, and mode of loading. However, most of these devices, not only commercial devices but also lab-special devices tailored to experimental need, are short of direct strain monitor or feedback. Inaccurate strain control would decrease the repeatability and comparability of experimental results. To address this gap, a strain feedback compensation method is proposed in this paper for accurate strain control.

The strain feedback compensation method needs an accurate measurement to monitor strain. The commonly used strain measuring methods about membranes involve the marker tracking measurement, the resistance strain gauge measurement, and the finite element analysis. In the marker tracking measurement [6], several predefined markers on the specimen are used to track the position change and thereby compute the strain. The accuracy of this method completely depends on the distribution of markers. Therefore, it is often regarded as a rough measuring technique. The resistance strain gauge measurement [8] is a basic strain measurement, but it is not suitable for the cell tensile experiments. On the one hand, the membrane is a kind of soft material so that the use of any contact-type sensor would affect its movement. On the other hand, this method is not able to measure the full-field strain distribution. The finite element analysis is commonly used in biomechanical researches. Many researchers [9–11] have used this method to compute the strain field of membranes. However, the finite element analysis is a theoretical calculation based on the models under ideal assumptions. It cannot be used to monitor actual strain during tensile experiments. Therefore, we need a highly accurate and contactless full-field strain measuring technique.

The digital image correlation (DIC) method is a widely used optical measuring technique [12]. Since it was first proposed [13, 14] in the 1980s, DIC has been extensively used in many fields [15–18]. Comparing to other strain measuring techniques, DIC has several advantages, such as fewer requirements in experimental environment, the absence of contact with specimen, and its high accuracy. In recent studies, many researchers focused on applying DIC to monitor the strain of biological tissues [19–24] and made great progress. Therefore, DIC is very suitable for monitoring the strain during the tensile experiments.

Thus, we proposed a strain feedback compensation method based on DIC to accurately control the strain of membranes during the tensile experiments. Five silicon rubber membranes with artificial speckle pattern were used as stretching specimens. Different strains ranging from 5% to 20% were conducted to the membranes. DIC was used for strain measurement during the whole process of strain feedback compensation at each strain level. The strain measured by DIC was then used as the parameter to compute the necessary compensation distance. Finally, we compared the strains measured before and after compensation with the target strains to evaluate the effect of our method.

## 2. Materials and Methods

### 2.1. Experimental Set-Up

The material of our membrane specimens is silica gel plate (DONGGUANSHI ZHISHENG RUBBER PRODUCTS Co. LTD), which is colorless and transparent. Therefore, the first step is to create the speckle pattern artificially. Lionello et al. [25] summarized several methods for creating speckle pattern on biological soft tissue. In this paper, the speckle pattern was made by spraying black and white quick-drying paints from an aerosol can. The white paint was first sprayed on the specimen to obtain a white background. Then, the black paint was sprayed on the specimen to obtain random black spots with a high contrast. In order to generate a fine mist of paints to ensure a uniform and random distribution of small paint dots, the distance between the nozzle and the specimen was maintained at more than 0.5 m [5]. Through these steps, the high-contrast speckle pattern suitable for DIC analysis was made. The size of the membrane specimens was 25 mm × 160 mm (thickness of 0.4 mm).

**Figure 1:**
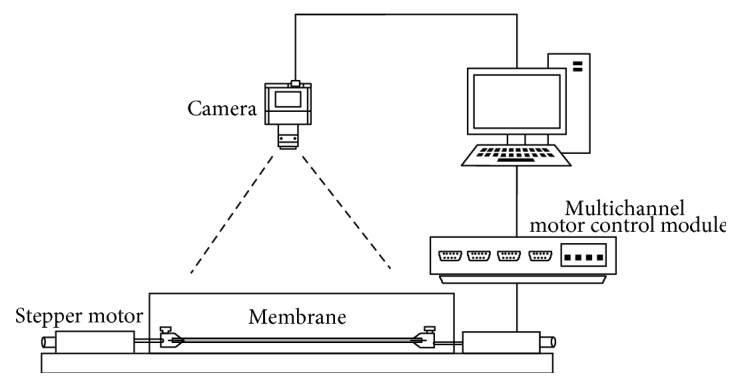
Experimental set-up.

As depicted in Figure 1(), the membrane was placed between two stainless steel clamps. During the experiments, an industrial camera (JHSM1400f, Shenzhen Jinghang Technology Co. Ltd.) was used for image capture. The images were recorded at the selected resolution of 2048 × 1536 pixels. Two stepper motors fixed on an optical platform were used to perform uniaxial stretching. A self-designed multichannel motor control module was used to control these stepper motors. A computer equipped with a 4-port RS-232 USB-to-serial converter (UPort 1450, MOXA) was programmed to provide the accurate synchrony of stepper motors via RS-232 serial ports and the image capture via USB. The acquired images were then processed with the DIC algorithm to calculate the strain field of the membrane.

### 2.2. Digital Image Correlation

**Figure 2:**
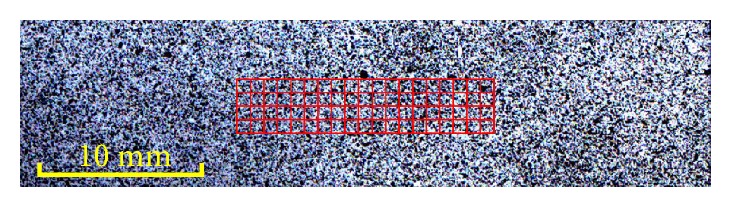
Selected region of interest (red rectangle).

DIC is an optical-numerical full-field measuring technique with subpixel accuracy. The method measures the displacement and strain field by tracking the same points (or pixels) between the two images recorded before and after deformation [12]. The image captured before and after stretching is called the reference image and deformed image, respectively. In general, the process of DIC involves three main steps. First, a region of interest (ROI) should be defined, which is further divided into evenly spaced virtual grids. As shown in Figure 2, the red rectangle shows the ROI and virtual grids. The intersection points of the virtual grids are selected as the center point of each subset. The subset is the matching unit in the DIC algorithm. Next, the reference image and deformed image should be reconstructed by a certain kind of subpixel registration algorithm to further improve the accuracy of DIC. In this paper, the fourth-order keys interpolant (Keys 4) algorithm [26] is used as the subpixel registration algorithm because it has small bias error and negligible rotation error. Finally, a correlation criterion should be defined to evaluate the degree of similarity between the reference and deformed subsets. Pan [27] provided an overview of various correlation criteria used in DIC. In this paper, the zero-normalized sum of squared differences (ZNSSD) was used as the correlation criterion because of its insensitivity to offset and linear scale in illumination lighting. Through the matching process based on ZNSSD, every reference subset would find its target subset in the deformed image, and the displacement and strain field could be obtained.

### 2.3. Strain Feedback Compensation Method

In order to calculate the compensation distance during tensile experiments, the compensation equations of uniaxial tensile stretching are proposed in this paper:

(1) $$\begin{array}{c} \frac{D_{t}}{S_{t}}=\frac{D}{S_{a}}, \end{array}$$

(2) $$\begin{array}{c} D_{t}=D+D_{c}, \end{array}$$

(3) $$\begin{array}{c} D_{c}=\frac{D}{S_{a}}S_{t}-D, \end{array}$$

where *D*_*t*_ is the necessary moving distance of the clamps to achieve the desired strain, *D* is the existing distance of the clamps, *S*_*a*_ is the actual strain measured by DIC, *S*_*t*_ is the desired target strain, and *D*_*c*_ is the necessary compensation distance. Equation (1) is based on the assumption that the stretching of the membrane remains in its mechanical linear region. Under the assumption, there is a linear relationship between the clamps' moving distance and the membrane's actual strain. Therefore, the ratio of *D*_*t*_ to *S*_*t*_ is equal to the ratio of *D* to *S*_*a*_. Because the sliding between membrane and the clamps exists, *D*_*t*_ is not equal to *D*. As shown in (2), *D*_*t*_ is comprised of *D* and *D*_*c*_. Substituting (2) into (1), the compensation equation (3) can be obtained, with which, the compensation distance *D*_*c*_ can be calculated.

**Figure 3:**
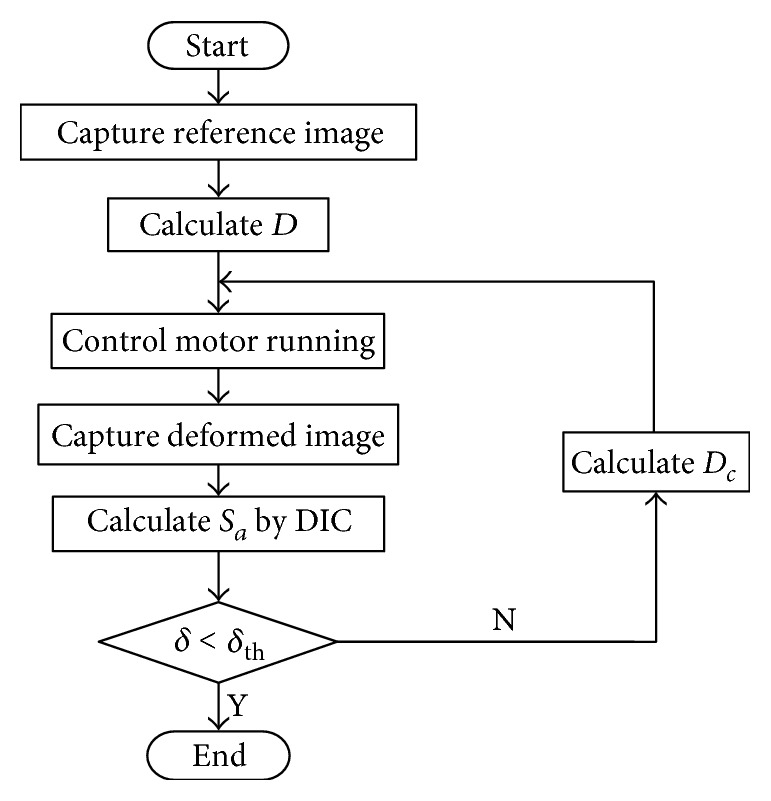
Flowchart of the strain feedback compensation process.

The detailed process of the strain feedback compensation is shown in Figure 3(). First, the image of membrane before stretching should be captured as the reference image. According to *S*_*t*_ and the initial clamping length of the membrane, *D* can be calculated and then used to control the stepper motors. This step is routine in traditional tensile experiments. After stretching, the image of stretched membrane should be captured as the deformed image. By inputting reference image and deformed image into the DIC program, *S*_*a*_ of the membrane can be calculated. Next, *S*_*a*_ should be compared with *S*_*t*_ to determine whether to perform the strain compensation. The judgment criterion is denoted by *δ*, and the threshold is denoted by *δ*_th_. In practice, *δ* can be set as an absolute error or relative error according to the experimental requirements. In this paper, *δ* represents the relative error between the actual strains and the target strains and *δ*_th_ is set to be 0.5%. If *δ* is greater than *δ*_th_, which means that the strain control does not satisfy the accuracy requirement, it is necessary to perform the strain compensation. According to (3), the compensation distance *D*_*c*_ can be calculated and then used to control the motors to complete compensation. Through the strain feedback compensation method, the strain can be accurately controlled.

## 3. Results and Discussion

**Figure 4:**
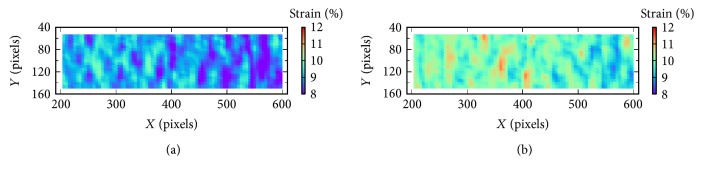
Strain filed of 5# specimen calculated by DIC before (a) and after (b) compensation at the target strain of 10%.

**Figure 5:**
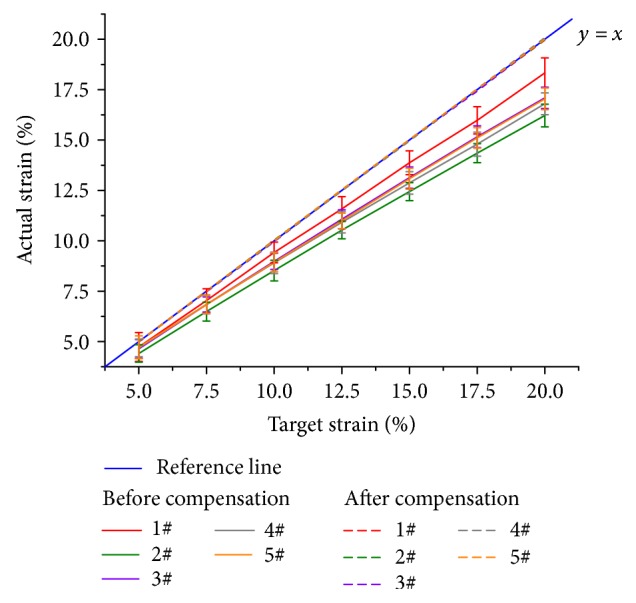
Strain control comparison. The solid lines represent the “before compensation” group, and the dashed lines represent the “after compensation” group. The error bars of the “after compensation” group are not shown.

**Figure 6:**
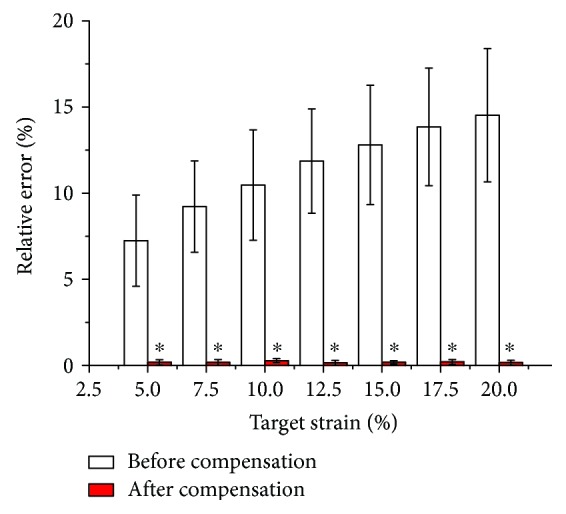
Relative error comparison at each strain level. Asterisk (^∗^) denotes statistically significant difference relative to the corresponding “before compensation” group at *P* < 0.01.

In order to evaluate the effect of our proposed strain feedback compensation method, a series of tensile experiments were performed on five specimens at a large strain range from 5% to 20% in increments of 2.5%. The actual strains of all specimens at each strain level were measured by DIC. The strains measured before and after compensation were called the “before compensation” group and the “after compensation” group, respectively. The statistical analysis was performed using *t*-test (*P* < 0.01) at each strain level. The results of all specimens are shown in Figures 4–6.

Figure 4 shows the strain filed of 5# specimen calculated by DIC before and after compensation at the target strain of 10%. As shown in Figure 4, the strain distribution of both is generally uniform, indicating that the membrane is homogeneous. In addition, most strain values before compensation (see Figure 4(a)) concentrate on 9%, while those after compensation (see Figure 4(b)) concentrate on 10%, demonstrating that the strain compensation method affects the entire stretching region and makes the whole strain field closer to the target value.

All strain results measured before and after compensation are shown in Figure 5. The horizontal axis represents the target strains, and the vertical axis represents the actual strains measured by DIC. Different colors represent different specimens. The solid lines represent the “before compensation” group, and the dashed lines represent the “after compensation” group. In Figure 5, the actual strains of the “before compensation” group show a strong correlation (*r* = 0.999) with the target strains. This result demonstrates that all specimens remained in their mechanical linear region during the tensile experiments, satisfying the assumption of our strain feedback compensation method. The blue solid line is the reference line (*y* = *x*), which represents an ideal situation of strain control that the actual strains equals to the target strains. As shown in Figure 5, the solid lines always stay below the reference line and all specimens show the same trend, which means that the errors before compensation mainly result from the systemic errors. Moreover, the dashed lines almost coincide with the reference line, which means that our strain compensation method is very effective.

The relative error comparisons at each strain level are shown in Figure 6. All the relative errors of the “after compensation” group are significantly lower than those of the “before compensation” group, showing statistically significant difference (*P* < 0.01). The relative error of the “before compensation” group increases with the increment of target strains. It is also notable that no matter what the target strain is, the relative error after compensation is always below 0.5%, which demonstrates that the effect of our strain feedback compensation method is very significant.

As mentioned above, the errors before compensation mainly result from systemic errors. Before compensation, the actual strains all stay below the reference line in Figure 5. Yet, the moving distance of the clamps is accurately controlled. That is to say, the clamp-to-clamp strains are always greater than the actual measured strains, indicating that the sliding of clamps is a nonnegligible error source. In this study, the clamped regions of the specimens were marked before clamping and stretching, and the sliding phenomenon was observed. It is known that the sliding is strongly related to the friction force between objects in contact. To moderate the sliding effect and thus reduce the error, the friction force between the clamps and specimen should be increased. In tensile experiments, the friction force between the clamps and specimen is determined by the clamping force and the friction coefficient of the contact surface. In this study, the slops of the five solid lines in Figure 5 are different, indicating that their sliding effects are different. However, the membrane properties and the clamps are the same among five experiments. The only differences among them are their clamping forces, operated manually, which cannot ensure the same. These results also demonstrate that the clamping force would affect the sliding effect. The clamping force cannot be too small or too large. Too small clamping force cannot ensure reliable clamping while too large force would damage the membrane, a kind of soft material, leading to unexpected change of mechanical properties of the membrane. However, to the best of our knowledge, there are few studies on how to choose the clamping force in tensile experiments. Besides choosing an appropriate clamping force, increasing the friction coefficient is also a good way to increase the friction force. In previous studies, some approaches were used to strengthen the fixation between specimens and clamps. Gao and Desai [20] utilized the cyanoacrylate, a kind of super glue, to fix the specimen. To minimize the sliding effect of the specimen, Karimi et al. [22] used a pair of coarse sandpaper glued to the lower and upper grippers. King et al. [28] fabricated specific serrated friction grips for tensile tests. Although these methods reduce the sliding effect to some extent, the additional modification of clamps restricts their application.

The proposed method in this paper is independent of the clamps and mechanical stretching instruments. The actual measured strain during stretching is used as feedback to compensate the strain error resulting from sliding. The results in our experiments demonstrate that our method is able to realize accurate strain control using existing stretching instruments without additional modification of clamps. Although the clamping forces for the five specimens cannot stay the same in this study, with our compensation method, the actual strain of the specimens all reach to the expected accuracy at every strain level. In the future, more work can be done in analyzing the fixation and clamping force in cell tensile experiments. However, before the sliding problem is totally solved, the strain monitor and feedback is suggested in tensile experiments in order to ensure the reliability and repeatability of the experiments.

## 4. Conclusions

In order to achieve accurate strain control, a strain feedback compensation method based on DIC is proposed in this paper. This method is independent of mechanical stretching instruments and can provide accurate strain feedback to help users monitor the strain condition. The compensation distance can be calculated by the proposed compensation equations. To evaluate the effect of our proposed method, a series of stretching experiments in different strains ranging from 5% to 20% were performed. The results showed that the proposed method can significantly decrease the strain errors and improve the experimental accuracy. Accurate strain control will allow researchers to design more complex and precise experiments and enhance the reliability and comparability of the test results.
